# Obesity Increases Disease Activity of Norwegian Patients with Axial Spondyloarthritis: Results from the European Map of Axial Spondyloarthritis Survey

**DOI:** 10.1007/s11926-020-00917-4

**Published:** 2020-06-23

**Authors:** Christian Bindesbøll, Marco Garrido-Cumbrera, Gunnstein Bakland, Hanne Dagfinrud

**Affiliations:** 1 Novartis Pharma AG, Oslo, Norway; 2 Health & Territory Research (HTR), Universidad de Sevilla, Seville, Spain; 3 Spanish Federation of Spondyloarthritis Associations (CEADE), Madrid, Spain; 4 Department of Rheumatology, University Hospital of Northern Norway, Tromsø, Norway; 5 National Advisory Unit on Rehabilitation in Rheumatology, Diakonhjemmet Hospital, Oslo, Norway

**Keywords:** Axial spondyloarthritis, Obesity, Body mass index, Disease activity

## Abstract

**Objective:**

To investigate the prevalence of overweight and obesity, as well as the association between body mass index (BMI) and disease activity in patients with axial spondyloarthritis (axSpA).

**Methods:**

Norwegian axSpA patients from the European Map of Axial Spondyloarthritis (EMAS) survey were included in this analysis. Sociodemographic, anthropomorphic, and disease-related variables (HLA-B27, comorbidities, BASDAI, and self-reported spinal stiffness) were reported. Patients were categorized into under/normal weight (BMI < 25 kg/m^2^), overweight (BMI ≥ 25 to < 30 kg/m^2^), and obese (≥ 30 kg/m^2^).

**Results:**

Of the 509 participants in the EMAS survey, 35% were categorized as under/normal weight, 39% overweight, and 26% obese. Compared to under/normal-weight patients, overweight patients had significantly higher degree of spinal stiffness (mean (SD) 7.91 ± 2.02 vs 7.48 (2.15) and number of comorbidities (2.45 ± 2.11, vs 1.94), both *p* < 0.001. Obese patients had significantly higher disease activity (BASDAI mean (SD) 5.87 ± 1.78 vs 4.99 ± 2.08, *p* < 0.001), degree of spinal stiffness (8.18 ± 2.03 vs 7.48 ± 2.15, *p* = 0.006), and number of comorbidities (3.43 ± 2.43 vs 1.94. ± .38, *p* < 0.001) than under/normal weight patients. After adjusting for gender and age, obesity proved to be independently associated with disease activity.

**Conclusion:**

Obesity was associated with higher reported BASDAI score, and being overweight or obese was associated with a higher degree of spinal stiffness and number of comorbidities compared to under/normal weight respondents. The results highlight the serious impact of obesity on health status, and obesity should therefore be considered as a modifiable risk factor for disease activity within the disease management of axSpA.

**Electronic supplementary material:**

The online version of this article (10.1007/s11926-020-00917-4) contains supplementary material, which is available to authorized users.

## Introduction

Axial spondyloarthritis (axSpA) is a chronic inflammatory rheumatic disease that primarily affects the axial skeleton [1]. The axSpA diagnosis comprises radiographic axSpA (r-axSpA), which corresponds to ankylosing spondylitis (AS), and non-radiographic axial spondyloarthritis (nr-axSpA). The disease is characterized by early onset, as patients experience their first symptoms in their twenties. The most typical clinical features are reduced spinal mobility, stiffness, and inflammatory low back pain [1]. Current treatment recommendations for people with inflammatory arthritis underline the importance of appropriately dosed physical activity to improve cardiorespiratory fitness and muscle strength [2, 3], but still, people with axSpA are shown to be less physically active and have lower physical fitness compared to the general population [4, 5].

Recent evidence from population-based studies points toward a combined effect of physical activity and body weight on mortality and health profile [6]. Increased weight is known to exert general biological stress, and a link between obesity and autoimmunity is suggested [7]. The exact mechanisms are not known, but evidence supports an association between increased levels of pro-inflammatory cytokines and adipokines derived from adipose tissue [7]. Knowledge about how obesity influences disease activity in axSpA patients is, however, limited. A small study reported more functional limitations and higher subjective disease activity and reduced the benefit of exercise in obese compared to normal weight AS patients [8]. Furthermore, obesity is also reported to be associated with impaired clinical outcome and reduced response to treatment with biological medication [9, 10]. Overweight axSpA patients are reported to show significantly lower response to tumor necrosis factor (TNF) inhibitors than normal-weight patients [10–12]. This knowledge adds to the existing large body of evidence of the negative health effects of excessed weight [13].

The aim of this study was to investigate the prevalence of overweight and obesity and to explore the association between body mass index (BMI) and disease activity, in Norwegian patients with axSpA included in the European Map of Axial Spondyloarthritis Survey [14••].

## Methods

### Design of Survey

The design and survey development of EMAS was recently reported [14••]. In brief, EMAS was a cross-sectional survey of unselected 2846 patients self-reporting axSpA from 13 different European countries: Austria, Belgium, France, Germany, Italy, the Netherlands, Norway, Russia, Slovenia, Sweden, Switzerland, the UK, and Spain. This analysis was based on data from the 509 Norwegian respondents. A Norwegian Patient Support Group, Spafo, supported recruitment by distributing the survey to its members. The questionnaire was completed via an online platform for survey data collection.

The patient questionnaire included information about sociodemographic variables (age, gender, educational level, marital status, employment status, income level, member of a patient association for axSpA), anthropomorphic (BMI), comorbidity, living habits (smoke and alcohol), and disease-related variables described below. BMI was classified according to WHO Europe. BASDAI was used to assess patient-reported disease activity. BASDAI includes six questions (Q) addressing fatigue/tiredness (Q1), neck, back or hip pain (Q2), pain/swelling in joints other than the neck (Q3), back or hips, discomfort of any areas tender to touch or pressure (Q4), morning stiffness from time of awakening (Q5), and duration of morning stiffness from time of awakening (up to 120 min) (Q6). Mean values for each of the six questions (Q1–6) and total BASDAI score (sum of Q1–Q4 + mean of Q5 and Q6, divided by 5) were calculated from 0 (no activity) to 10 (maximum activity) [15].

### General Stiffness Index

This index, developed specifically for EMAS, assessed the self-reported degree of stiffness experienced by patients in the cervical, dorsal, and lumbar areas of the spinal column. Possible responses range from least to most affected column and total scores are obtained by adding together the responses in each of the areas of the spine, resulting in a scale ranging from 3 (low degree of stiffness) to 12 (high degree of stiffness). This index showed acceptable internal reliability (Cronbach alpha = 0.73 for the Norwegian cohort) [14••].

### Comorbidities

The respondents’ self-reported comorbidities, including sleep disorders, anxiety, depression, obesity/overweight, hypertension, hypercholesterolemia, fibromyalgia, severe infections requiring antibiotics, psoriatic arthritis, cardiac arrhythmia, spinal or other fractures, cataracts, gout, diabetes, Crohn’s disease, severe infections requiring hospital admission, atherosclerosis, genital lesions, liver disease, and episcleritis.

### Sample Selection and Recruitment

Sample selection inclusion criteria were age ≥ 18 years, resident of Norway, self-reported diagnosis of axSpA (including ankylosing spondylitis, non-radiographic axSpA, and axSpA), and visit a healthcare professional for axSpA in the 12 months prior to participation.

### Statistics

Sociodemographic and disease-specific variables are presented as means and standard deviations (SD). BMI was categorized into three groups (under/normal, overweight, and obese). BASDAI was categorized into two groups: high (≥ 4) or low (< 4) disease activity. Chi-square test (*χ*^2^) was used to check independence between two categorical variables by means of a contingency table. The Mann-Whitney *U* test (2 groups) or Kruskal-Wallis *H* test for independent samples (> 2 groups) was used to test group differences. The BASDAI scores (the index and the sub-scales) for the different BMI categories were visualized in bar graphs. The association between BMI and BASDAI was tested with a Pearson correlation test. The level of statistical significance was set at *p* < 0.05.

## Results

### Sociodemographics and Disease Characteristics

Out of the 509 Norwegian participants with axSpA, 69.7% were women. The mean age was 48 ± 12 years, 55.2% had education at the university level, 74.9% were married, and 60.1% were members of a patient support group. Most axSpA patients (75.1%) did not smoke and 50.3% reported never or occasionally drink alcohol (Table 1).

**Table 1:** Sociodemographic, anthropometric characteristics, and lifestyle habits

Variable, *n* patients with data available	Mean ± SD/*n* (%)
Age (years) *n* = 509	48 ± 12
Gender (female), *n* = 509	355 (69.7)
Marital status, *n* = 509	
Single	67 (13.2)
Married	381 (74.9)
Separated/divorced	53 (10.4)
Widowed	8 (4.6)
Educational level, *n* = 509	
No schooling completed	1 (0.2)
Primary school	26 (5.1)
High school	201 (395)
University	281 (55.2)
BMI, *n* = 509	
Under/normal weight (< 25)	180 (35.4)
Overweight (25–29.9)	189 (39.1)
Obesity (> 30)	130 (26.5)
Smoking, *n* = 509	
Non-smoker	382 (75.1)
Less than 10 cigarettes/day	59 (11.6)
More than 10 cigarettes	68 (13.4)
Alcohol consumption, *n* = 509	
Never or occasionally	256 (50.3)
1–2 times per week	214 (42.0)
More than twice per week	39 (7.7)
Member of a patient support group, *n* = 2846	360 (60.1)

In total, 35.4% (*n* = 180) of the participants were under/normal weight (BMI < 25), 39.1% (*n* = 199) were overweight (BMI 25–20), and 26.5% (*n* = 130) were obese (BMI > 30), resulting in 65.6% of the respondents in this study categorized as overweight or obese (Table 1). Only six of 180 patients (3.3%) were underweight in the under/normal weight group.

The majority of participants reported a diagnosis of AS (66.4%), while the remaining reported being diagnosed with nr-axSpA (12.6%) or just axSpA without specifying the subtype (21%) (Table 2). The mean disease duration was 22.9 ± 12.7 years. The majority of the tested patients were HLA-B27 positive (82.3%). The participants had on average more than two comorbidities. The most commonly reported comorbidities were sleep disorder, hypertension, and depression (Supplementary Table 1). The mean BASDAI score was 5.3 ± 2.0 and the majority of the patients (74.7%) were classified into the high disease activity group (≥ 4). The BASDAI score was higher in females (5.5 ± 1.9) than in males (4.9 ± 2.0). The mean (SD) score of spinal stiffness was 7.8 ± 2.1, reported on a scale with a range from 3 (low) to 12 (high) degree of spinal stiffness.

**Table 2:** Disease-specific characteristics in Norwegian axSpA patients

Variable, *n* patients with data available	Mean ± SD/*n* (%)
Type of condition, *n* = 509	
Ankylosing spondylitis	338 (66.4)
Non-radiographical axial spondyloarthritis	64 (12.6)
Unspecified axial spondyloarthritis	107 (21.0)
Disease duration (years), *n* = 509	5.3 ± 2.0
HLA-B27 (tested), *n* = 503	351 (69.8)
Positive	289 (82.3)
Negative	62 (17.7)
Number of reported comorbidities, *n* = 499	2.51 (2.4)
Spinal stiffness index (3–12), *n* = 509	7.83 ± 2.01
BASDAI (0–10), *n* = 509	5.3 ± 2.0
Females	5.5 ± 1.9
Males	4.9 ± 2.0
BASDAI cutoff, *n* = 509	
< 4	129 (25.3)
≥ 4	380 (74.7)

### Disease Variables in Under/Normal Weight, Overweight, and Obese axSpA Patients

**Fig. 1:**
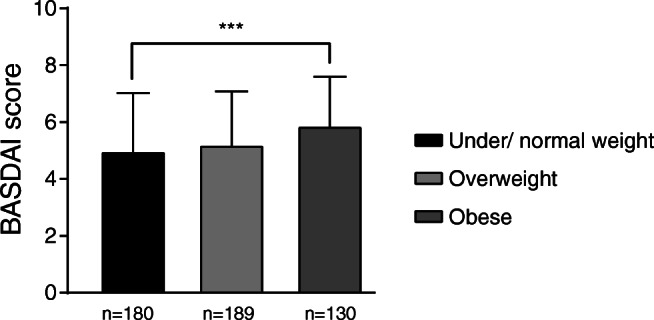
Disease activity in under/normal weight, overweight, and obese patients. Patients were classified into groups based on BMI as described in the “Methods” section. The number of patients in each group is indicated under the representative bar. Obese patients reported higher disease activity as compared to normal weight. All values are mean ± SD. ***p < 0.001 by Student’s *t* test

**Fig. 2:**
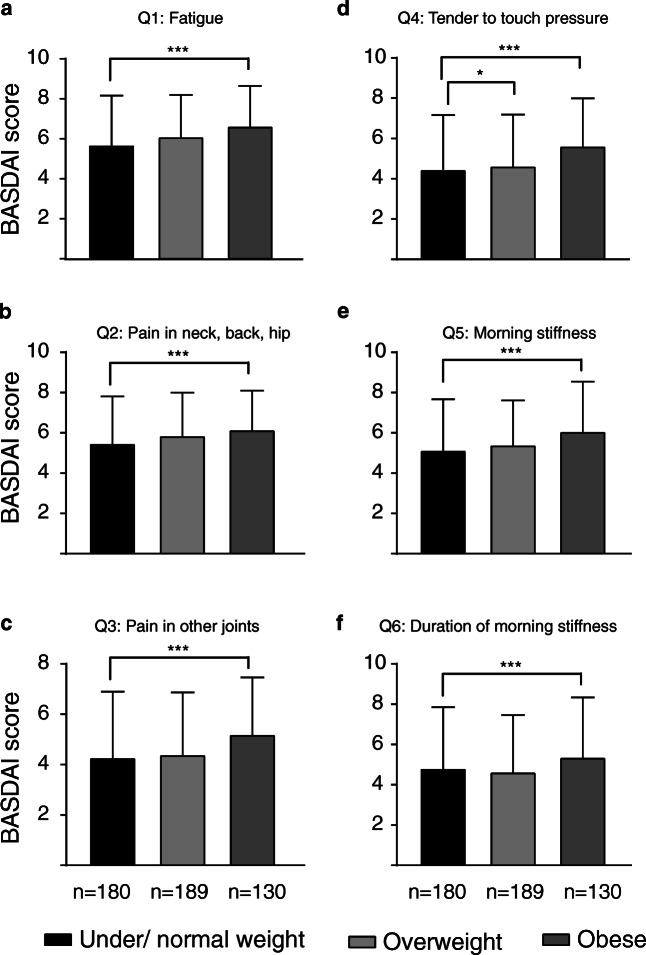
Disease activity as shown by individual BASDAI questions in under/normal weight, overweight, and obese patients. Patients were classified into groups based on BMI as described in the “Methods” section. The number of patients in each group is indicated under the representative bar. Obese patients reported higher scores on all six individual BASDAI questions (Q1–Q6) related to disease activity as compared to normal weight. All values are mean ± SD. *p < 0.05, ***p < 0.001 by Student’s *t* test

To study associations between weight and disease activity, we compared disease variables in groups of under/normal weight, overweight, and obese patients. Being overweight or obese was associated with a higher degree of spinal stiffness and number of comorbidities compared to normal weight respondents (Table 3). The average BASDAI score was significantly higher in obese than in under/normal-weight patients (5.87 ± 1.78 vs 4.99 ± 2.08, *p* < 0.001) (Table 3, Fig. 1). Obese patients reported significantly higher scores on all six questions in the BASDAI scale compared to under/normal-weight patients (Fig. 2 and Table 3). The association between BASDAI and BMI was significant when adjusting for gender and age in a linear regression analysis (*p* = 0.001).

**Table 3:** Disease-specific characteristics in under/normal weight, overweight, and obese Norwegian axSpA patients. All values are mean ± SD. *p < 0.05, ***p < 0.001 by Student’s *t* test

	Under/normal weight	Overweight	Obese
BASDAI (index)	4.99 (2.08)	5.21 (1.92)	5.87 (1.78)***
Q1	5.73 (2.50)	6.10 (2.16)	6.64 (2.07)***
Q2	5.48 (2.40)	5.86 (2.20)	6.15 (2.01)***
Q3	4.28 (2.67)	4.40 (2.52)	5.20 (2.32)***
Q4	4.46 (2.70)	4.64 (2.61)*	5.63 (2.43)***
Q5	5.14 (2.60)	5.40 (2.28)	6.07 (2.54)***
Q6	4.82 (3.10)	4.64 (2.89)	5.37 (3.03)***
Spinal stiffness (3–12)	7.48 (2.15)	7.91 (2.02)***	8.18 (2.03)***
Comorbidities (*n*)	1.94 (2.38)	2.45 (2.11)***	3.43 (2.43)***

## Discussion

The results of this study indicate a higher burden of disease in overweight or obese patients compared to under/normal-weight patients with axSpA. Obese patients reported worse scores on all subscores as well as the total score of the patient-reported disease activity index (BASDAI). Furthermore, being overweight or obese was associated with a higher degree of spinal stiffness and number of comorbidities compared to normal weight respondents. There was also seen a significant association between BASDAI and BMI (higher BASDAI with higher BMI) when adjusted for gender and age.

According to large population-based Norwegian studies, 60–70% of adults are either overweight or obese, and the minority is normal weight [16, 17]. In the Norwegian EMAS population, 65% of the respondents were categorized as overweight or obese, with a mean BMI of 27.4. Even if this is similar to the BMI in the general Norwegian population [16, 17], the finding is of clinical importance, as adipose tissue is known to release pro-inflammatory cytokines. Increased BMI, especially increased abdominal fat, may therefore potentially add to the inflammatory burden in patients with systemic inflammatory diseases.

The two main drivers of overweight and obesity are unhealthy diet and physical inactivity. The direct evidence of effects of diet as a disease modifier in rheumatic diseases is scarce, but a meta-analysis of six randomized controlled trials addressed the effects of weight management, showing that weight loss could prevent the onset of psoriasis and improve pre-existing psoriasis in obese individuals [18••]. Furthermore, promoting a healthy diet is also important as overweight patients are reported to have a poorer response to medication, such as TNFα inhibitors [9]. Thus, a healthy diet and weight control should be addressed in consultations with patients at risk for developing a rheumatic disease as well as in patients with established disease, treated with relevant medication.

A healthy diet and physical activity are the most important factors in achieving a beneficial body composition. Physical inactivity leads to the accumulation of visceral fat mass and increased abdominal fat and is associated with impaired glucose and lipid metabolism as well as higher production of pro-inflammatory cytokines (adipokines), as for example, interleukin-6 (IL-6) and TNFα [19]. Furthermore, also the skeletal muscles contribute to an anti-inflammatory milieu, and physical inactivity has been shown to be associated with an increased level of pro-inflammatory muscle markers (IL-1β, IL-6) in rheumatoid arthritis [20, 21]. Thus, the amount of adipose tissue together with the level of skeletal muscle activity probably plays important roles in the balance of pro- and anti-inflammatory cytokines [19].

Overweight or obesity may also represent a biomechanical factor that can be considered as a trigger of the inflammatory process that may influence the pathogenesis of SpA in terms of new bone formation. This hypothesis was investigated in an animal study, concluding that mechanical strain may drive both entheseal inflammation and new bone formation [22]. In line with this, the effect of obesity on radiological outcomes in patients with SpA was summarized in a recent systematic review, concluding that higher BMI was associated with the formation of syndesmophytes and enthesophytes, as well as with more radiographic manifestations [23••]. The increased radiographic changes could probably explain the reported effect of BMI on clinical outcomes [11].

Weight control and physical fitness are well-documented pathways toward health and disease control [24], but Pinto et al. state that the role of these factors as disease modifiers in inflammatory rheumatic diseases are overlooked [25••]. In addition to the strong link between inactivity and cardiovascular disease, which is prevalent comorbidity following these diseases [26, 27], it is also growing evidence for the anti-inflammatory effect of high-intensive exercise. The theoretical rationales for these mechanisms are thoroughly presented [28, 29], and meta-analyses and clinical effect studies have demonstrated that intensive exercise is well tolerated and may reduce disease activity in axSpA patients [30, 31].

We acknowledge the data from the EMAS study has some limitations. The survey depended on self-reported data and did not attempt to confirm participant diagnosis nor to support participant responses with clinician-reported assessments. Accordingly, clinical data including BASDAI and spinal stiffness score may also suffer from response bias. Nevertheless, the sample characteristics were consistent with previous cohorts including patients with confirmed axSpA [32–35].

The medical treatment options have revolutionized the field of rheumatology in the last decades, and many patients experience reduced disease burden as a result of effective biological medication [30, 31, 36]. Still, the axSpA patients in the EMAS cohort report the negative impact of overweight and obesity on disease activity. Therefore, health professionals seeing patients with axSpA should address weight control as an important element of disease management.

## Electronic Supplementary Material

ESM 1 (PDF 918 kb)
