# Supplementary material for: Obesity Increases Disease Activity of Norwegian Patients with Axial Spondyloarthritis: Results from the European Map of Axial Spondyloarthritis Survey
Source: Curr Rheumatol Rep. 2020 Jun 23;22(8):43. doi: 10.1007/s11926-020-00917-4 (PMC7311492; doi:10.1007/s11926-020-00917-4)
Supplement: Supplementary file 1 — (PDF 918 kb) [file 11926_2020_917_MOESM1_ESM.pdf]

Supplementary table 1

Comorbidities	n (%)
Sleep disorders	174 (35.9)
Hypertension	115 (23.3)
Depression	108 (21.6)
Obesity / Overweight	90 (18.4)
Hypercholesterolemia	78 (16.0)
Fibromyalgia	71 (14.6)
Severe infections requiring antibiotics	71 (14.3)
Anxiety	71 (14.3)
Psoriatic arthritis	67 (14.0)
Cardiac arrhythmia	58 (11.9)
Spinal or other fractures	40 (8.1)
Cataracts	31 (6.3)
Gout	23 (4.7)
Diabetes	23 (4.6)
Crohn's disease	21 (4.3)
Severe infections requiring hospital admission	21 (4.2)
Atherosclerosis	18 (3.7)
Genital lesions	12 (2.4)
Episcleritis	11 (2.4)
Liver disease	10 (2.0)
Glaucoma	10 (2.0)
Heart failure	10 (2.0)
Kidney failure	7 (1.4)
Coronary artery disease	7 (1.4)
Pacemaker fitted	3 (0.6)
